# Structure–mechanics relationships of collagen fibrils in the osteogenesis imperfecta mouse model

**DOI:** 10.1098/rsif.2015.0701

**Published:** 2015-10-06

**Authors:** O. G. Andriotis, S. W. Chang, M. Vanleene, P. H. Howarth, D. E. Davies, S. J. Shefelbine, M. J. Buehler, P. J. Thurner

**Affiliations:** 1 Institute for Lightweight Design and Structural Biomechanics, Vienna University of Technology, Getreidemarkt 9, Vienna 1060, Austria; 2 Bioengineering Research Group, Faculty of Engineering and the Environment, University of Southampton, Southampton SO17 1BJ, UK; 3 Department of Civil Engineering, National Taiwan University, Taipei 10617, Taiwan, Republic of China; 4 Department of Bioengineering, Imperial College London, London, UK; 5 The Brooke Laboratories, Division of Infection, Inflammation and Immunity, Faculty of Medicine, University of Southampton, Southampton SO16 6YD, UK; 6 Department of Mechanical and Industrial Engineering, Northeastern University, Boston, MA, USA; 7 Center for Materials Science and Engineering, Massachusetts Institute of Technology, Cambridge, MA, USA; 8 Center for Computational Engineering, Massachusetts Institute of Technology, Cambridge, MA, USA; 9 Laboratory for Atomistic and Molecular Mechanics, Department of Civil and Environmental Engineering, Massachusetts Institute of Technology, Cambridge, MA, USA

**Keywords:** collagen, structure, mechanics, osteogenesis imperfecta, atomic force microscopy, atomistic simulations

## Abstract

The collagen molecule, which is the building block of collagen fibrils, is a triple helix of two α1(I) chains and one α2(I) chain. However, in the severe mouse model of osteogenesis imperfecta (OIM), deletion of the COL1A2 gene results in the substitution of the α2(I) chain by one α1(I) chain. As this substitution severely impairs the structure and mechanics of collagen-rich tissues at the tissue and organ level, the main aim of this study was to investigate how the structure and mechanics are altered in OIM collagen fibrils. Comparing results from atomic force microscopy imaging and cantilever-based nanoindentation on collagen fibrils from OIM and wild-type (WT) animals, we found a 33% lower indentation modulus in OIM when air-dried (bound water present) and an almost fivefold higher indentation modulus in OIM collagen fibrils when fully hydrated (bound and unbound water present) in phosphate-buffered saline solution (PBS) compared with WT collagen fibrils. These mechanical changes were accompanied by an impaired swelling upon hydration within PBS. Our experimental and atomistic simulation results show how the structure and mechanics are altered at the individual collagen fibril level as a result of collagen gene mutation in OIM. We envisage that the combination of experimental and modelling approaches could allow mechanical phenotyping at the collagen fibril level of virtually any alteration of collagen structure or chemistry.

## 1. Introduction

### 1.1. Collagen: molecular and fibrilar structure

Collagen is a family of proteins that compose approximately 30% of the protein mass of the human body [1]. This makes collagen one of the most abundant proteins in humans as well as vertebrates and consequently, perhaps, the most important protein family providing structural and mechanical stability to almost every biological tissue in our bodies.

**Figure 1.**
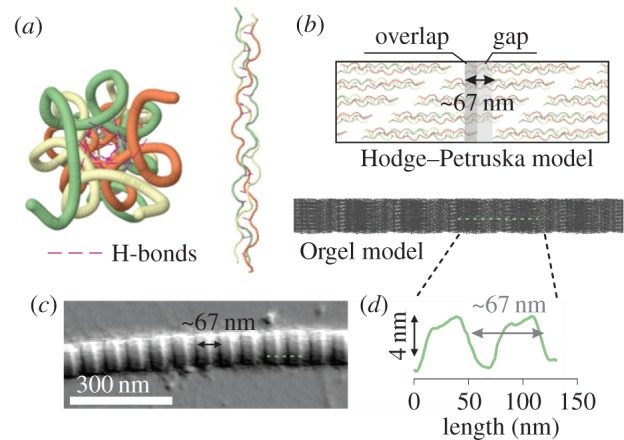
(*a*) Front view and side view (tilt away from the reader) of a collagen molecule showing the right-handed twist of the triple helix as well as the hydrogen bonds present to stabilize the triple helix (Jmol; collagen structure from Protein Data Bank; PDB 1Cag). (*b*) Self-assembly of molecules into a collagen microfibril with the characteristic D-periodicity of 67 nm as a result of overlap and gap regions. (*c*) Atomic force microscopy image of a collagen fibril and (*d*) height topography profile graph showing the D-periodicity.

The characteristic structure of collagen molecules has been proposed over 50 years ago on the basis of X-ray diffraction [2]. At the lowest length-scale level, three polypeptide alpha (*α*) chains are closely packed into a right-handed twisted helix (figure 1*a*; collagen structure from Protein Data Bank; PDB 1Cag [3]). The triple helix has two small non-helical domains (telopeptides) located at each end of the molecule. The close packing into a triple helix is mediated by the high content of glycine (Gly) residues [4]. Gly occupies every third position in the amino acid sequence of each α-chain and is always positioned in the core of the triple helix [5,6]. This results in the motif (Gly-X-Y), where X and Y are occupied by other amino acids [7]. Finally, stabilization of the triple helix into a collagen molecule is mediated by hydrogen bonds [8] (magenta coloured dashed lines in figure 1*a*) as well as hydroxylation of the proline and lysine residues [9], formed between the α-chains.

The triple helical formation takes place intracellularly; but once the molecules are exported outside the cell, they self-assemble into long fibrils by lateral and longitudinal organization. Seen in electron micrograph images in the transverse direction of the fibrils, there are regions where the density of molecular packing is decreased (known as gap regions; figure 1*b*), and regions where the density of the molecular packing is increased (known as overlap regions; figure 1*b*) [10,11]. The gap and overlap regions occur periodically along the length of the fibril and their D-periodicity is 67 nm [11]. Petruska & Hodge [12] suggested a simplified two-dimensional model (shown in figure 1*b*) of the lateral staggering of the molecules resulting in the 67 nm periodicity. Several other models that try to explain the D-banding exist, but the most recently proposed and commonly used is a super-twisted three-dimensional microfibril structure, which is characterized as a triclinic unit cell [13].

### 1.2. The role of intermolecular cross-linking in collagen

The type of collagen cross-links are divided into (i) enzymatic and (ii) non-enzymatic cross-links, depending on the origin of their formation. The enzymatic cross-links are first to form during the fibrilar assembly of collagen, and are administered by cells [14,15]. In particular, enzymes, such as lysyl oxidase and hydroxylase, act on specific molecular segments deaminating the lysine and hydroxylysine residues located at the telopeptides of the collagen molecule [16]. This results in the formation of a divalent Schiff base intermolecular cross-link between the non-helical and helical domains of adjacent molecules [17]. These divalent cross-links mature over time into trivalent bonds connecting a third adjacent molecule [17]. These cross-links play a significant role in stabilizing the fibrilar structure and are responsible for stiffening of the tissue [18]. Non-enzymatic cross-links result from the instantaneous chemical reactions between collagen and glucose [17]. The process initiates with a Maillard reaction, which is a reaction between amino acids and sugar [19]. Further oxidation leads to the formation of advanced glycation end products (AGEs). The non-enzymatic cross-links can form anywhere between the helical domains of adjacent collagen molecules [17]. Stiffening of the arterial wall with naturally increased AGEs [20] as well as stiffening of the Achilles' tendons of rats [21] with artificially increased AGEs have been observed. This clearly suggests the role of AGEs in the mechanical properties of biological tissues. But, AGEs are also suggested to play a significant role in the development of collagen-related pathologies, such as diabetes mellitus (type II), kidney pathology, osteogenesis imperfecta (OI) and cardiovascular pathology [22].

### 1.3. Collagen mutation related to structure–mechanical changes

The mechanical importance of collagen manifests itself by a range of collagen gene mutations resulting in pathologies, such as Ehlers–Danlos syndrome [23], Alport syndrome [24] and OI [25]. These pathologies are characterized by disorders of the connective tissue. In this context, one of the most detrimental clinical phenotypes of OI is brittle bones [25].

Brittle bones, as well as other clinical phenotypes of OI [25], result from mutations in the COL1A1 and COL1A2 genes [26]. These genes encode the type I procollagen α1 and α2 chains and such mutations result in non-functional polypeptide alpha chains in collagen type I [27]. In OI, single Gly substitution by other bulkier amino acids in the α-chains [28] impairs the folding of the triple helix [29]. The impaired folding of the OI collagen molecule manifests itself via micro-unfolded regions at physiological temperatures [30]. Such impairments result in severe variants of OI in humans (types II and III) [31,32] with an abnormally increased number of bone fractures and defects in collagen-rich tissues [33].

To study the effects of OI mutations on the structure–function of biological tissues a number of mouse models exist that simulate the clinical phenotypes of OI [34]. The mouse model system that simulates moderate–severe phenotypes of OI, i.e. the homozygous oim mice or OIM, results from complete deletion of the COL1A2 gene [34,35]. Deletion of COL1A2 results in the absence of α2(I) chains and OIM model form homotrimeric type I collagen molecules, because of the substitution of α2(I) chain by a third α1(I) chain [36].

**Figure 2.**
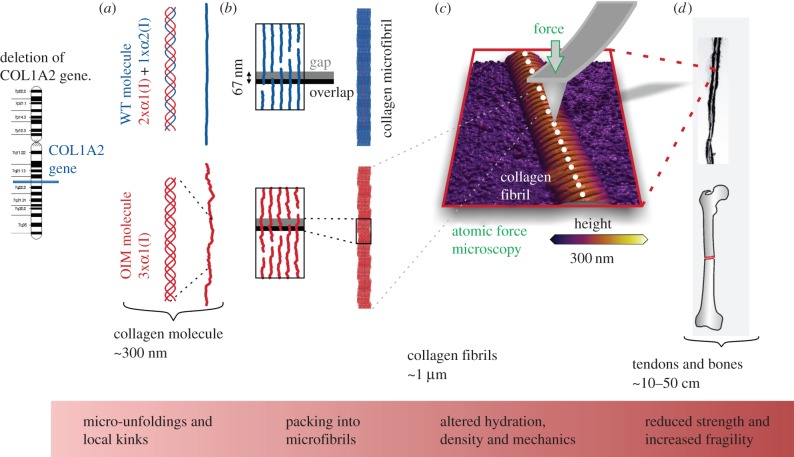
A bottom-up view of the physical alterations of collagen fibrils synthesized from the OIM mouse model of osteogenesis imperfecta. Deletion of the COL1A2 gene results in the absence of α2(Ι) chains in the OIM collagen molecule. (*a*) Absence of α2(Ι) chains results in homotrimeric molecules preventing close packing of the three chains into collagen molecules and resulting in micro-unfoldings and local kink formations at the molecular level of OIM collagen. (*b*) This alters the packing of molecules into microfibrils having detrimental effects on (*c*) hydration (this study), density and mechanical properties at the fibril level. (*d*) Eventually, collagen-rich tissues such as tendons and bones have reduced strength and increased fragility.

Synthesis of homotrimeric collagen molecules causes a number of structural, mechanical and biochemical alterations, summarized in figure 2, that transmit throughout the structural hierarchy of collagen-rich tissues such as bones and tendons. The substitution of a single *α*-chain results in structural changes similar to those in single-point mutations. Recently, Chang *et al.* show that local kink formations accompanied by micro-unfolded regions characterize the structure of the type I homotrimeric collagen molecule [37] (figure 2*a*). Micro-unfolded regions and local kinks result in lower packing of these molecules into collagen fibrils (figure 2*b*) [38]. Structural impairment at smaller length scales are responsible for knock-on effects on larger length scales, summarized in figure 2*c,d*, in terms of structure and mechanics [39].

In the case of the OIM, both the structure and mechanics are altered at the bone tissue and organ level [40,41]. Yet, while OIM bones have been characterized and found to be brittle at the whole bone level [42–44], the structural and mechanical changes at the individual collagen fibril level and below have so far been little [41] or not at all experimentally characterized. Both theoretical, such as molecular dynamics [45,46], and experimental approaches, such as atomic force microscopy (AFM) [47], enable assessment of these length scales. Molecular dynamics focus on the single molecule scale [48] as well as the mesoscale levels [49] of collagen fibrils. The experimental mechanical assessment of individual collagen fibrils, with diameters ranging from 30 to 500 nm [50,51], is accomplished, among other techniques, with AFM in force mode [47,52–55].

We hypothesize that the structure and mechanical properties are altered at the fibril level of OIM collagen, because of changes in the primary structure of collagen molecules and as a consequence also of the packing of these molecules into fibrils. To answer this hypothesis, we undertook a comparative study of the structure and mechanics of collagen fibrils from the OIM and wild-type (WT) mice. This was accomplished by employing AFM imaging and cantilever-based nanoindentation in both hydrated and dehydrated environments. For interpretation of experimental results, we compared these with predictions of mechanical performance obtained via molecular dynamics simulations. Most importantly, our results show that the transverse stiffness of collagen fibrils is dependent on collagen phenotype and in turn on the amount of bound water that can be taken up by the fibrils. Additionally, we show that the absence of α2(Ι) chains in the OIM collagen fibrils leads to lower levels of unbound water, which affects the mechanical properties at this length-scale level and effectively, leads to stiffer collagen fibrils when fully immersed in an aqueous solution.

## 2. Material and methods

### 2.1. Chemicals

Phosphate-buffered saline (PBS) tablets were obtained from Sigma Aldrich, UK. Ethanol (EtOH; 99.5% v/v) was obtained from Fisher Scientific. Ultrapure deionized water (Milli-Q DIRECT 5) was used for all experiments.

### 2.2. Animals and collagen sample preparation

Five-month-old B6C3Fe-a/a-+/+ WT and pathologic B6C3Fe-a/a-Col1a2*Oim*/*Oim* mice were culled; tails were dissected and stored in gauze soaked in PBS (pH = 7.4) at −18°C until further use. A tendon section was harvested from each mouse tail using scalpel and tweezers. Each tail tendon section was directly deposited on a poly-l-lysine coated microscope glass slide (Thermo Scientific). To reveal areas with individual collagen fibrils the tail tendon sections, while still hydrated, were smeared out on the glass slide surface with the use of scalpel and tweezers similarly as in [47]. Samples were air-dried and stored in a desiccator until further use.

### 2.3. Atomic force microscopy

The AFM experiments were carried out in a temperature (20.7 ± 0.7°C) and humidity (47.9 ± 6.7%) controlled room with a MFP-3D atomic force microscope (Asylum Research, Santa Barbara, CA, USA). A standard open fluid cell (Fluid Cell Lite, Asylum Research) was used to perform experiments in aqueous and EtOH solutions.

#### 2.3.1. Atomic force microscopy imaging

Unless otherwise specified, AFM images of individual collagen fibrils were obtained in contact mode at approximately 1 Hz scanning rate in air on dehydrated samples and in PBS solution, before indentation testing. For swelling measurements, i.e. increase in collagen fibril height from the dehydrated to the fully hydrated state (in PBS), the same collagen fibrils were imaged subsequently before and after hydration.

#### 2.3.2. Atomic force microscopy cantilever-based nanoindentation

AFM cantilever-based nanoindentation tests were performed in fully hydrated samples (PBS), in air-dried and in two PBS solutions with an increasing concentration of EtOH (25% and 50% concentration of EtOH in PBS solution). For nanoindentation tests on fully hydrated samples (PBS as hydration medium), the V-shaped (PNP-TR, NanoWorld AG, Switzerland) and rectangular (PNP-DB, NanoWorld AG, Switzerland) pyrex nitride cantilevers of (0.24 ± 0.02) N m^−1^ and (0.35 ± 0.05) N m^−1^ spring constant, respectively (nominal values are 0.32 N m^−1^ and 0.48 N m^−1^ for PNP-TR and PNP-DB, respectively), with a silicon nitride pyramidal tip of less than 10 nm tip radius were employed. For nanoindentation tests in air-dried samples, silicon cantilevers of (44.5 ± 5.6) N m^−1^ spring constant (nominal value is 40 N m^−1^) with trihedral silicon tips of less than 10 nm tip radius (NSC15 rectangular cantilevers, MicroMasch) were used. For nanoindentation tests in EtOH, a silicon cantilever with spring constant (8.3 ± 1.8) N m^−1^ and approximately 7 nm tip radius (AC200, Olympus) was used. The spring constant of each AFM cantilever was determined with the thermal method [56].

**Figure 3.**
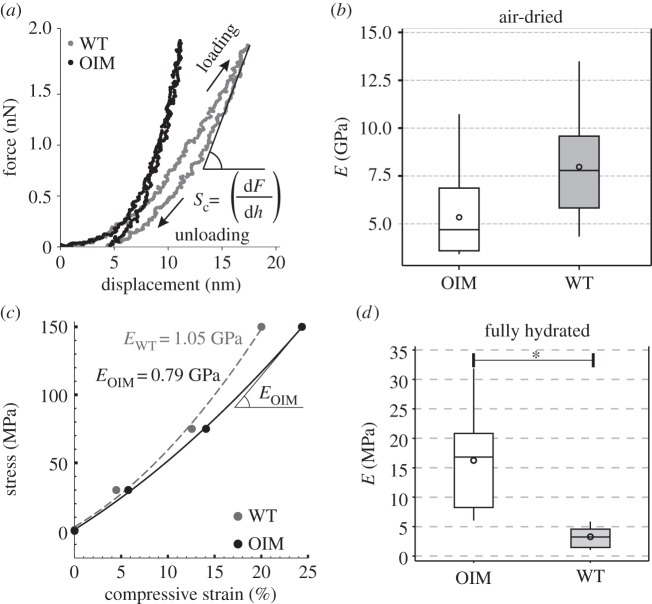
(*a*) Representative experimental force–displacement curves from AFM cantilever-based nanoindentation on fully rehydrated OIM (black) and WT (grey) collagen fibrils. (*b*) Box plots of indentation modulus of air-dried collagen fibrils. OIM collagen fibrils show a 33% lower (*p < 0.05) indentation modulus compared with the WT collagen fibrils. (*c*) Compressive stress–strain curves of WT (grey) and OIM (black) collagen microfibril models. These models simulate the experiments in air-dried conditions and similarly show a difference between the elastic moduli values (25% lower elastic modulus in OIM microfbrils). (*d*) Box plots of indentation modulus of hydrated OIM and WT collagen fibrils. The average indentation modulus of hydrated OIM collagen fibrils is significantly higher compared with the WT fibrils (*p < 0.001).

Both dry as well as hydrated collagen fibrils were tested similar to a recent study [50]. In brief, the AFM tip apex (approx. 10 nm in radius) situated at the free end of an AFM cantilever (with known stiffness) indents the surface of the collagen fibrils resulting in force–displacement curves (electronic supplementary material, §S1) shown in figure 3*a*.

### 2.4. Determination of indentation modulus

The reduced modulus of two elastic bodies in contact is given by the equation [57]:2.1
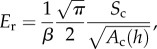
where *β* is a dimensionless parameter which is close to unity and varies with the indenter shape (1.0226 < *β* < 1.085) [57]. As previously suggested [50], we have taken *β* equal to 1. The reduced modulus *E*_r_ of two elastic bodies in contact is a function of the Poisson's ratios of the two bodies and their elastic moduli [58]:2.2
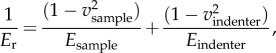
where *v*_sample_ and *v*_indenter_ the Poisson's ratio of the sample and indenter, respectively, and *E*_sample_ and *E*_indenter_ the elastic modulus of the sample and the indenter, respectively. The elastic modulus of silicon [59] AFM tip is much larger than the elastic modulus of the collagen fibrils whether hydrated or dried (*E*_indenter_ = 169 GPa where *E*_sample_ for collagen ranges from 5 MPa to 13 GPa). In the case of 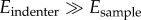 equation (2.2) takes the form [47]:2.3
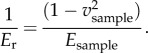


By substitution of equation (2.3) in equation (2.1), we calculate the elastic modulus of the sample from the equation:2.4
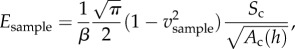
with *A*_c_ the projected area function and *S*_c_ the contact stiffness. Determination of the projected area function for the AFM tips we have used is described in §S2 of the electronic supplementary material as well as in detail [50].

The analysis approach described above assumes that the two bodies in contact are elastic and isotropic. However, a collagen fibril is characterized by a transverse isotropic structure as ultra-long collagen molecules, 300 nm in length and 1.5 nm in width, are aligned parallel to the longitudinal axis of the collagen fibril [60,61]. The structural anisotropy influences the mechanical properties such as stiffness, which are different if measured in the transverse and longitudinal direction of the collagen fibrils [13,47]. Indentation loading is not considered as uniaxial and, therefore, due to the structural anisotropy of collagen fibrils, the resulting elastic modulus, from the experiments presented in this study, will rather be a contribution of the compressive transverse and the tensile longitudinal elastic modulus. We, therefore, choose to term the elastic modulus of collagen fibrils as the indentation modulus when measured via indentation type loading.

### 2.5. Swelling measurements of collagen fibrils

Collagen fibrils swell upon hydration in an aqueous salt solution [53]. Swelling was measured as per fold increase in height of the collagen fibrils upon hydration (electronic supplementary material, §S3). In total, eight WT and seven OIM collagen fibrils were imaged before and after hydration in PBS. Based on the findings from the experiments and from our *in silico* study, we estimated the normalized density of collagen fibrils as a function of swelling. The estimation is presented in the electronic supplementary material, §S4.

### 2.6. Chemical dehydration with ethanol

The OIM and WT collagen fibrils were mechanically assessed also during chemical dehydration with EtOH, to investigate the effect of dehydration on the mechanics as well as fibril diameter. Two experiments were performed in total.

The first experiment was performed by initially testing both samples in PBS in a fluid cell. Subsequently, the concentration of EtOH was gradually increased at 25% per volume by adding EtOH in the existing PBS solution, without removing the sample from the AFM stage. We noted that further addition of ethanol, above 25% per volume, resulted in the formation of bubbles on the AFM cantilever as well as across the sample surface (possibly due to gas exchange). At this point, the second experiment was performed.

In the second experiment, two solutions of ethanol mixed in PBS were prepared; one with 25% and the other with 50% EtOH. After initially testing the sample in PBS, the solution was completely removed from the fluid cell and the second solution was then added. This procedure was repeated until the samples were tested in 100% EtOH while avoiding bubble formation.

A further study was performed to measure the shrinking of collagen fibrils during chemical dehydration. This trial was performed in a NanoWizard^®^ ULTRA Speed A AFM system (JPK Instruments AG, Berlin) by employing PNP-DB cantilevers (0.48 N m^−1^ spring constant). One collagen fibril per condition (OIM and WT) was subsequently imaged in air, PBS and EtOH (25%, 50% and 100%).

### 2.7. Statistics

All statistical analyses were performed in Minitab^®^ 16.2.4. The generalized linear model (GLM) was used to test differences between unbalanced groups. The natural logarithm was applied to correct for unequal variances. Group comparisons were based on the least square mean (lsmean) values. Tukey's test was used to account for multiplicity. Results were considered to be significant for *p* < 0.05. Unless otherwise specified, data are presented as arithmetic mean ± s.d.

### 2.8. Collagen microfibril model

To gain further understanding of the experimental results and to determine the structure–mechanics relationship at the air-dried state of collagen fibrils atomistic simulations were performed on collagen microfibril models composed of healthy and pathologic collagen molecules. The collagen microfibril model was generated based on the *in situ* structure of full-length type I collagen molecule [13] (Protein Data Bank identification code 3HR2), which has a triclinic unit cell (*a* ≈ 40.0 Å, *b* ≈ 27.0 Å, *c* ≈ 678 Å, *α* ≈ 89.2°, *β* ≈ 94.6°, *γ* ≈ 105.6°). Note that the structure reported in [13] includes only backbone *α* carbons and the primary sequence of *Rattus norvegicus*. We, therefore, used homology modelling, described in [62], to obtain a full-atomistic structure with the *Mus* collagen sequence. The real sequences of type I α1(I) and type I α2(I) chains of *Mus musculus* (WT mouse) were used to match the collagen fibrils tested in experiments. The heterotrimer collagen microfibril model was composed of two α1(I) chains and one α2(I) chain, whereas the homotrimer collagen microfibril model was composed of only α1(I) chains. Sequences were adopted from the NCBI protein database (http://www.ncbi.nlm.nih.gov/protein): AAH50014.1 for α1(I) chain and NP-031769.2 for α2(I) chain. In both collagen microfibril models, ions were added to neutralize the system. Full-atomistic simulations were performed using modelling code LAMMPS [63] (http://lammps.sandia.gov/) and the CHARMM force field [64] that includes parameters for hydroxyproline amino acids, based on a model put forth by Anderson [65]. An energy minimization using a conjugate gradient scheme was performed prior to molecular dynamics simulations. Rigid bonds are used to constrain covalent bond lengths and an integration time step of 1 fs was used. Non-bonding interactions were computed using a cut-off for neighbour list at 13.5 Å, with a switching function between 10 and 12 Å for van der Waals interactions. The electrostatic interactions were modelled by the Ewald/n style, which performs standard coulombic Ewald summations in a more efficient manner [66]. After energy minimization, the collagen molecule was simulated through 5 ns at a constant temperature of 310 K in molecular dynamics simulations to obtain an equilibrium structure. In order to assess the mechanical properties of the dehydrated atomistic microfibril models, which mimic the experimental environment of indentation, we performed molecular dynamics simulations with four different constant mechanical stress levels (1.013 bar, 30 MPa, 75 MPa and 150 MPa) in compression along the fibril *a*-axis while maintaining a constant 1.013 bar on the other axes. The stresses were applied for 5 ns and molecular dynamics simulation for each load applied. We used the last 1 ns of molecular dynamics simulation for computing the *a*-axis unit cell lattice, corresponding to each load applied.

## 3. Results

### 3.1. Indentation modulus of air-dried collagen fibrils

Box plots of the indentation modulus of OIM and WT collagen fibrils measured in the air-dried condition (bound water present) are shown in figure 3*b*. The indentation modulus of the air-dried OIM collagen fibrils is *E*_OIM-Air_ = (5.3 ± 2.2) GPa and of the WT-collagen fibrils, it is *E*_WT-Air_ = (7.9 ± 2.8) GPa. In air, the indentation modulus of OIM collagen fibrils is significantly lower (*p* < 0.05) compared with the WT collagen fibrils.

### 3.2. Atomistic simulation results

To simulate the experimental indentation testing, compression loading was applied along the transverse direction of a collagen microfibril model. Compressive loading in WT and OIM collagen microfibrils results in a nonlinear elastic stress–strain relation (figure 3*c*). The stress–strain behaviour was characterized by polynomial functions 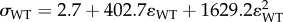 for WT and 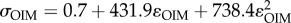 for OIM for four (4) different applied stresses of 1 atm, 30 MPa, 75 MPa and 150 MPa. The transverse elastic modulus is calculated by the slope of the stress–strain at a given stress level. At 150 MPa stress the transverse elastic modulus is *E*_OIM_ = 0.79 GPa for the OIM collagen microfibrils and *E*_WT_ = 1.05 GPa for the WT collagen microfibril (25% difference), which is in agreement with experimental results from fibril indentation conducted in air-dried samples.

### 3.3. Indentation modulus of OIM and WT collagen fibrils in phosphate-buffered saline

Box plots of the indentation modulus of hydrated collagen fibrils (PBS at pH 7.4) from the OIM and WT mouse tail tendons are shown in figure 3*d*. Groups with significant differences are indicated with an asterisk (*). The mean indentation modulus of the OIM collagen fibrils is *E*_OIM-PBS_ = (16.2 ± 3.0) MPa (*N* = 8 fibrils) and of the WT collagen fibrils is *E*_WT-PBS_ = (3.3 ± 0.5) MPa (*N* = 10 fibrils). In PBS, the indentation modulus of OIM collagen fibrils (lsmean = 14.2 MPa) is overall fivefold higher (*p* < 0.001), compared with the WT collagen fibrils (lsmean = 2.8 MPa; figure 3*d*). No statistical differences were observed between the indentation modulus when data were compared per gender (results shown in electronic supplementary material, §S5).

### 3.4. Swelling measurements of OIM and WT collagen fibrils

**Figure 4.**
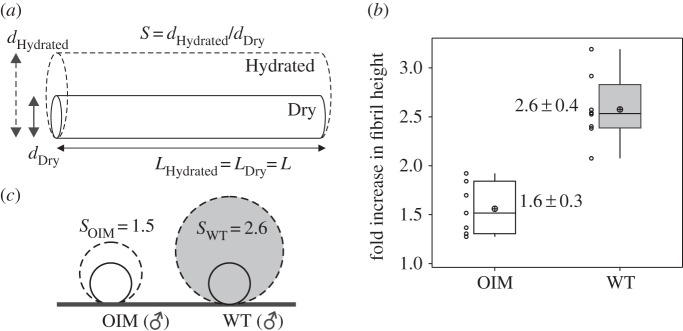
(*a*) Estimation of the swelling, *S*, of collagen fibrils, upon hydration in PBS, assuming that the length of the fibril does not change. (*b*) Box plots and individual values of the fold increase in height of WT and OIM collagen fibrils when hydrating with PBS from the air-dried state. The average fold increase in height is 2.6 ± 0.4 (*N* = 8 collagen fibrils) in (male) WT collagen fibrils and 1.5 ± 0.3 (*N* = 7 collagen fibrils) in OIM collagen fibrils. OIM collagen fibrils show a 42% statistically significant lower (*p*-value < 0.001) swelling (i.e. fold increase in fibril height) compared with the WT collagen fibrils. (*c*) Swelling in (male) WT and OIM collagen fibrils.

Figure 4*a* illustrates the estimation of swelling of collagen fibrils upon hydration in PBS. Box plots of the fold increase in height for OIM and WT collagen fibrils (male animals) are shown figure 4*b*. The fibril height of WT collagen fibrils increases 2.6-fold and of OIM collagen fibrils 1.5-fold, hence the swelling in OIM collagen fibrils is significantly lower (42%) compared with WT collagen fibrils (*p* < 0.001). The swelling from air-dried to fully hydrated state (immersed in PBS) as well as the difference in swelling between OIM and WT collagen fibrils is illustrated in figure 4*c*.

### 3.5. Effect of chemical dehydration on structure and mechanics

**Figure 5.**
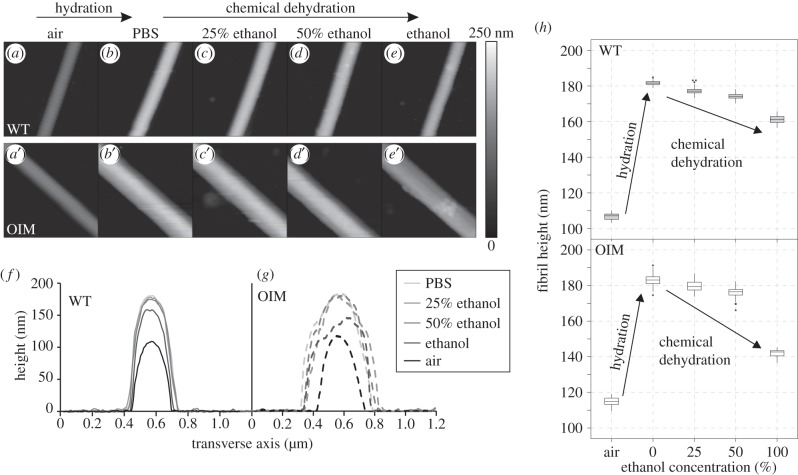
(*a–e*) AFM height topography images of a WT and (*a′–e′*) OIM collagen fibril during chemical dehydration. (*f,g*) Representative cross-section profiles of WT and OIM collagen fibrils, respectively, during hydration in PBS as well as chemical dehydration with ethanol. (*h*) Box plots of diameter of collagen fibrils upon hydration with PBS solution and dehydration with ethanol.

Figure 5 shows AFM height topography images of a WT (figure 5*a–e*) and OIM (figure 5*a′–e′*) collagen fibril recorded in air, PBS, 75% PBS (25% EtOH), 50% PBS (50% EtOH) and EtOH solution as well as representative cross-section profiles (figure 5*f,g*). Box plots of the fibril height upon hydration and chemical dehydration are shown in figure 5*h*. WT and OIM samples swell upon hydration and shrink with chemical dehydration. The OIM collagen fibrils swell less than the WT and shrink more when chemically dehydrated in ethanol (electronic supplementary material, figure S5*a*).

**Figure 6.**
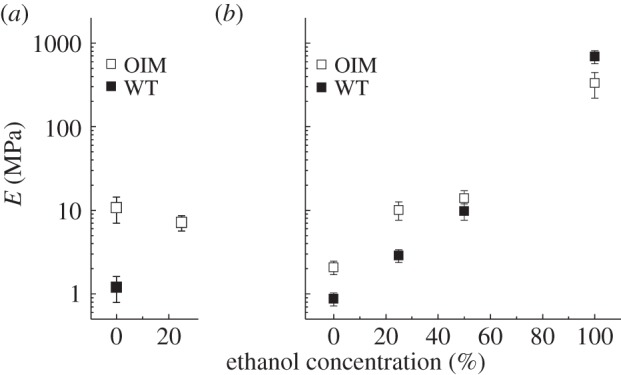
Results of indentation modulus from trial 1 (*a*) and trial 2 (*b*). Mean indentation modulus of an OIM and a WT collagen fibril measured at increasing concentrations of ethanol from fully hydrated state in PBS (i.e. 0% ethanol concentration). In both OIM and WT collagen fibrils, the indentation modulus increases by three orders of magnitude at 100% ethanol when compared with 100% PBS.

Further to structural changes during chemical dehydration, the mechanics are also altered. Figure 6 illustrates the effect of ethanol concentration (% per volume) on the indentation modulus. Results from the first trial show only the indentation modulus determined at the hydrated state in PBS (for both samples) and at 25% EtOH (for OIM only). Similar to the results presented in §3.2, the OIM indentation modulus (*E*_OIM_ = 10.8 ± 3.7 MPa) is significantly (approx. 9-fold) higher compared with the WT one (*E*_WT_ = 1.2 ± 0.4 MPa). Note that the same samples were tested in the second trial but the indentation modulus of the OIM samples dropped at the fully hydrated state between the two trials. While we may not have a full explanation why the indentation modulus of the OIM decreased in the second trial, we assume this may be because of non-reversible effects of the ethanol (due to the incubation in 25% EtOH in trial 1) on the structure and hence the mechanical properties of collagen fibrils. Importantly, results from the second trial show how the indentation modulus increases with the concentration of EtOH in the PBS solution. Although the OIM and WT indentation moduli increase by almost three orders of magnitude at 100% EtOH, the difference between OIM and WT indentation moduli decreases with increasing the EtOH concentration. Finally, when the collagen fibrils were tested in 100% EtOH, the OIM fibril shows, similarly to air-dried samples, a lower indentation modulus compared with the WT one.

## 4. Discussion

### 4.1. Structural alterations in OIM collagen fibrils

Structural and mechanical alterations of collagen are the hallmarks of OI, a collagen-related genetic disease [1]. The OIM mouse model represents an extreme case of this pathology [34], but the genetic modification (deletion of the COL1A2 gene resulting in homotrimeric collagen molecules) results in brittle bones highly characteristic of OI [40,67].

**Figure 7.**
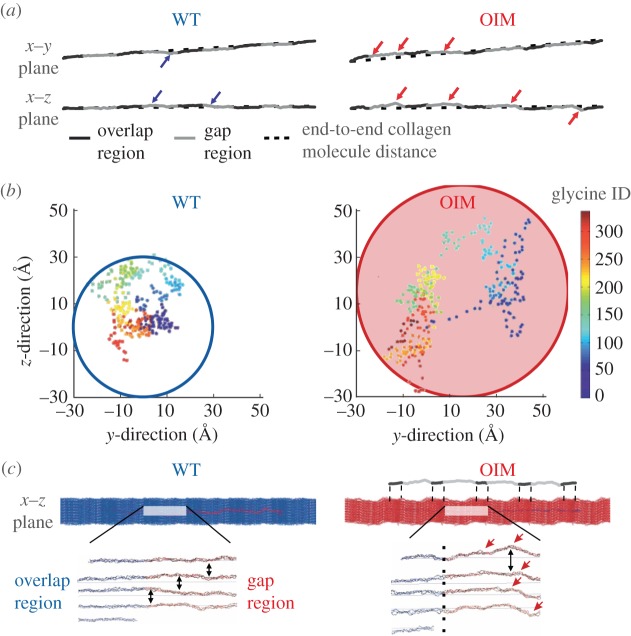
Structural differences between WT and OIM collagen molecules and microfibrils under compressive stress of 75 MPa. (*a*) *x*–*y* plane (defined by the *c* and *a* axes of the unit cell) and *x*–*z* plane (defined by the *c* and *b* axis of the unit cell) of WT and OIM collagen molecules while under stress of the collagen microfibril. (*b*) Centre of mass (COM) of glycine residues projected to the plane perpendicular to the end-to-end vector of the molecule. The colour indicates the ID (identification number) of the glycine residue from the left (blue) to the right (red) of the collagen molecule. The first and the last COM of glycine residues lies at the origin. (*c*) *x*–*z* plane of the WT (blue) and OIM (red) collagen microfibrils. The five transversely staggered collagen molecules are also presented below the collagen microfibrils models to illustrate the effect of larger local kink formations in OIM collagen molecules on their lateral packing.

Recent atomistic simulations revealed larger local kink formations in the OIM collagen molecule [37]. The larger local kinks resulted in approximately twofold lower persistence length in the OIM molecule compared with the WT one [37]. Local kink formations and the lower persistence length [36,38] in OIM collagen could explain the loss of lateral packing of molecules into fibrils depicted in our atomistic simulations as illustrated in figure 7*a*. Figure 7*a* compares kink formations (present study) between the WT and OIM collagen molecules while under compressive stress of 75 MPa. During compressive loading, local kinks are larger in the OIM collagen molecule compared with the WT one, as depicted in figure 7*b*. Figure 7*b* illustrates projections of the centre of masses of glycine residues to the plane perpendicular to the end-to-end vector of the molecule, tracking in this manner the three-dimensional conformation of the backbone of the molecule. Kink formations indicate impairment of collagen packing and lead to larger deviations of the position of glycine residues from the cross-sectional centres of mass.

At the microfibril level of collagen, our atomistic models show that at 1 atm (cf. electronic supplementary material, table S2), the OIM unit cell has larger ***a*** and ***b*** axis (***a***_OIM_ = 36.16 Å and ***b***_OIM_ = 25.54 Å) compared with the WT, i.e. normal (***a***_WT_ = 33.41 Å and ***b***_WT_ = 25.08 Å). Consequently, the OIM microfibril has about 8% and 2% larger ***a*** and ***b*** lateral axes, respectively, and therefore increased intermolecular spacing. In fact, our results suggest a 14% higher unit cell volume in the OIM microfibril. Assuming similar weights for the WT and OIM collagen (Uniprot; *Μ*_α1(I)_ = 138.032 kDa and *Μ*_α2(I)_ = 129.557 kDa, i.e. heterotrimeric: *M*_WT_ = 405.621 kDa and *M*_OIM_ = 414.096 kDa yielding to 2% difference), the OIM collagen microfibril has 12% lower density when dehydrated and by inference, this explains the lower compressive elastic modulus of the air-dried, i.e. where only bound water is present, OIM collagen microfibril (25% from *in silico* study). In agreement with results from atomistic models, our experiments also show a lower indentation modulus (33%) of the air-dried OIM collagen fibrils. In summary, local kink formations result because of the absence of α2(I) chains in the OIM collagen molecule. These local kinks act as steric barriers that block the close packing of the collagen molecule into a fibril (as illustrated in figure 7*c*). In addition, the less hydrophobic content of α2(I) [38,68] could further affect the close packing into fibrils because more bound water is present in the OIM collagen. The effect of hydration on the structural and mechanical changes in OIM collagen fibrils is discussed further in the following sections.

### 4.2. Impaired hydration and its effect on OIM collagen fibril mechanics

The importance of water in the mechanical properties of collagen fibrils manifests itself by the relative amount of water present in the fibrils. Upon hydration in PBS (bound and unbound water), we estimated that approximately two-thirds of the total volume of the hydrated collagen fibril is water. Hence, the mechanical properties of collagen fibrils highly depend on their interaction with intra-fibrilar water [53,69]. Here, both bound and unbound water need to be considered. The unbound water would be considered as the water content within a fibril that evaporates upon drying in air. Hence, bound water is present in the ‘dehydrated’ microfibril model as well as the air-dried collagen fibrils [69,70]. The amount of bound water influences collagen density, namely the equatorial diffraction spacing, which is related to the intermolecular spacing [71]. The equatorial diffraction spacing was found to increase by approximately 2 Å with increasing bound water content by approximately 0.4 g g^−1^ dry collagen, from X-ray diffraction measurements on air-dried rat tail tendon [70]. Moreover, based on estimations of volume fraction of water via differential scanning calorimetry, Miles *et al.* suggested a 7% increase in the intermolecular separation in the OIM collagen fibril while the volume fraction of bound water increased by approximately 6% [72]. Higher content of unbound water correlates with the intermolecular separation distance [70] and has been inversely related to the amount of intermolecular non-enzymatic cross-links [73]. Interestingly, the latter have no effect on the bound water content [73]. Recently, a higher amount of non-enzymatic cross-linking was reported for demineralized OIM compared with WT bones [41].

**Figure 8.**
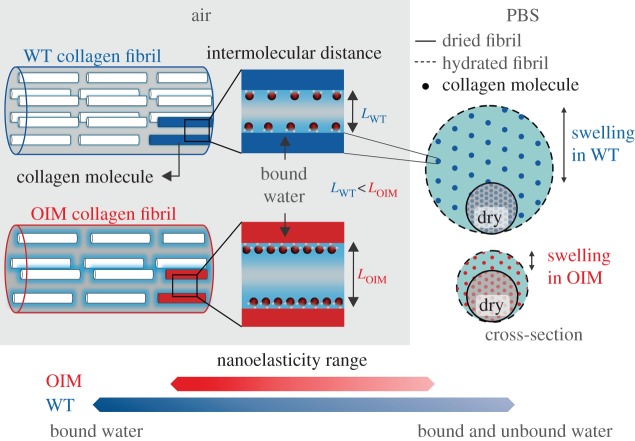
The structure–mechanical phenotype of collagen fibrils from the OIM mouse model. In air, the OIM collagen fibril has increased bound water resulting in a less dense and thus softer collagen fibril compared to the WT fibril. Upon hydration in phosphate-buffered saline, the OIM fibril swells less, allowing less unbound water to be incorporated in the structure of these fibrils. Owing to the lower swelling in OIM and the less dense structure, the range of indentation modulus that can be achieved in OIM is lower compared with WT collagen fibrils.

Therefore, we can summarize the most important suggested differences between OIM and WT type I collagen from previous studies as well as this one as follows: homotrimeric OIM collagen type I is more loosely packed, contains more bound water and has a higher amount of non-enzymatic cross-links. Upon immersion of OIM collagen fibrils in physiological buffer only a lower hydration with unbound water and hence smaller swelling can be achieved in OIM compared with WT collagen fibrils because of the higher non-enzymatic cross-linking and the higher content of bound water. This is summarized in a schematic in figure 8.

In fact our results show a marked, approximately twofold reduction in swelling of OIM collagen fibrils (cf. figure 4*b*) as well as a fivefold increase in the indentation modulus compared with WT collagen fibrils. The interpretation of this is that both bound and unbound water in collagen are due to the interaction of ionic side groups with water and salt ions, hence hydration is due to osmotic pressure generated on the collagen within the surrounding salt solution. Upon drying, this pressure disappears and the fibril contracts. The contraction pressure and the local affinity of water to collagen, determines how much water can be kept within a fibril and one would expect packing density to largely determine the contraction pressure. According to this, the lower packing density of OIM collagen can explain its higher amount of bound water. As already discussed, another reason for the differences in swelling, between OIM and WT collagen fibrils could be the higher non-enzymatic glycation of OIM collagen [41]. Our study does not allow us to deduce if one of these effects is dominating but probably they are both involved. Also we cannot offer explanations for the high level of non-enzymatic glycation of OIM collagen. We can only speculate that this may be a biological response to make up for a lower initial stiffness, i.e. before maturation and ageing occurs, or, simply because glycation is much more favoured in the OIM collagen. It is reasonable to assume, though, that local kink formations may expose more amino acids to glycation as opposed to normal collagen. Be that as it may, if these findings can be extended to human cases of OI, one can speculate that treatment with an AGE inhibitor, such as aminoguanidine, might reduce the glycation of collagen fibrils, which could be beneficial for the mechanical properties of collagen-rich tissues in such patients.

### 4.3. Effect of chemical dehydration

Bound water plays an important role in backbone structure stabilization by forming a layer of hydration around the collagen molecule [3,74,75]. Removing this layer of hydration results in conformational changes of the collagen molecule [75,76] and further shrinking of the collagen fibril.

During chemical dehydration at low concentrations of EtOH, the unbound water is removed. This results in decreasing intermolecular spacing [70] and therefore gradual shrinking of the collagen fibrils (cf. figure 5). As the concentration of ethanol increases, some bound water would be removed. Now ethanol will also cause some swelling, which is governed by the non-polar groups. Assuming that the interaction of ethanol with the non-polar groups of the collagen molecules results in an altered structural conformation of the molecules, it may be that some bound water, which was not accessible before any structural change, can now be removed. Because of the decreased amount of water but the increased ethanol concentration, the diameter of the collagen fibrils is less than that when fully hydrated but larger than in air-dried conditions (cf. figure 5*h*).

Shrinking of the collagen fibrils due to chemical dehydration is also expected to impact the mechanical properties. Our results show a drop in the indentation modulus of the OIM hydrated collagen fibrils by fivefold after the first trial of ethanol dehydration (cf. figure 6). Here, changes in mechanics may occur as a result from permanent conformational changes due to ethanol treatment. Interestingly, our results show different shrinking behaviour in the OIM compared with WT collagen fibrils (electronic supplementary material, figure S5*a*).

Following our line of argumentation the higher shrinking in OIM (during chemical dehydration) suggests a denser collagen fibril and therefore a higher indentation modulus compared with WT. But, our AFM mechanical assessment results actually show a lower indentation modulus (electronic supplementary material, figure S5*b*) for the OIM compared with the WT collagen fibril. The normalized density (electronic supplementary material, figure S5*b*) is an estimation based on the change of fibril diameter assuming no inter- and intra-molecular changes during chemical dehydration. Nevertheless, immersion in 100% EtOH leads also to structural changes in collagen and here OIM collagen might be more affected due to local kinks and unfolded regions. Therefore, the density estimations, especially for EtOH might carry substantial error. In this context, future tensile experiments on individual collagen fibril will allow a better estimation of the change in length upon dehydration.

## 5. Conclusion

AFM experiments as well as atomistic simulations show that collagen fibrils are characterized by altered hydration, structural properties and, hence, mechanical function as a consequence of deletion of COL1A2 gene. Owing to the higher bound water content in air-dried OIM collagen fibrils compared with WT ones, the intermolecular separation is larger, which is responsible for the lower indentation modulus, as also confirmed by our atomistic simulations. The situation is reversed upon full hydration, leading to a significantly higher indentation modulus of OIM collagen fibrils compared with WT ones. This higher indentation modulus is due to the lower swelling, i.e. the lower ability of the fibrils to absorb unbound water. A possible explanation why this ability is lowered in OIM fibrils is either the increased bound water content, leaving less room for unbound water, and/or the previously reported increase of non-enzymatic cross-linking resisting fibril dilation. Furthermore, we show that the accessible range of indentation moduli is lower in OIM collagen fibrils for the different environments observed in this study. Structural alterations in the OIM collagen fibrils may be responsible for the increased AGEs, previously reported. This could be explained by an increased number of exposed amino acids, as a result of local kink formations and micro-unfolded regions. If this finding can be extrapolated to human patients, one possible future course of action might be treatments similar to those applied in diabetes, i.e. the use of AGE inhibitors to counteract excessive AGEs.

## Supplementary Material

ESM document (figS1 to S4 and Table S1)

## Supplementary Material

Table S2-ESM

## Supplementary Material

Figure S5-chemical dehydration

## Data accessibility

Data are available in Figshare under the link http://figshare.com/s/1a3bf250e9c411e482d806ec4b8d1f61.
