# Supplementary material for: Structure–mechanics relationships of collagen fibrils in the osteogenesis imperfecta mouse model
Source: J R Soc Interface. 2015 Oct 6;12(111):20150701. doi: 10.1098/rsif.2015.0701 (PMC4614505; doi:10.1098/rsif.2015.0701)
Supplement: ESM document (figS1 to S4 and Table S1) [file rsif20150701supp1.pdf]

Electronic Supplementary Materials for:

Structure-mechanics relationships of collagen fibrils in the Osteogenesis Imperfecta Mouse model

O. G. Andriotis[1, 2], SW. Chang[3,4], M. Vanleene[5], P. H. Howarth[6], D. E. Davies[6], S. J. Shefelbine[5,7], M. J. Buehler[3,8,9], and P. J. Thurner[1,2,*]

1. Institute for Lightweight Design and Structural Biomechanics, Vienna University of Technology, Getreidemarkt 9, Vienna 1060, Austria.
2. Bioengineering Research Group, Faculty of Engineering and the Environment, University of Southampton, SO17 1BJ, Southampton, UK
3. Laboratory for Atomistic and Molecular Mechanics, Department of Civil and Environmental Engineering, Massachusetts Institute of Technology, Cambridge, Massachusetts, USA.
4. Department of Civil Engineering, National Taiwan University, Taipei 10617, Taiwan.
5. Department of Bioengineering, Imperial College London, London, United Kingdom
6. The Brooke Laboratories, Division of Infection, Inflammation and Immunity, University of Southampton, Faculty of Medicine, SO16 6YD, Southampton, UK.
7. Department of Mechanical and Industrial Engineering, Northeastern University, Boston, Massachusetts, USA
8. Center for Materials Science and Engineering and 9. Center for Computational Engineering, Massachusetts Institute of Technology, Cambridge, Massachusetts.

*To whom correspondence may be addressed at:
Prof. Philipp J. Thurner
Institute for Lightweight Desing and Structural Biomechanics
Vienna University of Technology
Getreidemarkt 9
A – 1060 Vienna
Austria
phone: +43 (1) 58801 31723
fax: +43 (1) 58801 - 317 99
E-mail: pthurner@ilsb.tuwien.ac.at

S1 Acquisition of force-displacement curves

Data acquisition in this study is based in a recently developed standardized methodology (Andriotis, Manuyakorn et al. 2014).

Briefly, an AFM image of about 10 µm x 10 µm is recorded to discriminate individual collagen fibrils lying on the stiff substrate from fibril bundles. Subsequently a number of collagen fibrils are then individually tested after performing another AFM image which includes one collagen fibril. Depending on the size of the collagen fibril the image scale ranges from 1 µm x 1 µm to 3 µm x 3 µm. AFM cantilever-based nanoindentation is then performed by Force Volume (FV) map. The FV map resolution ranges from 30 lines x 30 points to 50 lines x 50 points. the choice of the FV map resolution depends on that force-displacement curves must be recorded on the fibril crest, which is necessary to eliminate inaccuracy arising from invalid contact between the AFM tip and the collagen fibril due to the cylindrical fibril geometry (Andriotis, Manuyakorn et al. 2014). To allow user friendly data collection the AFM fast scan direction was aligned to the fibril longitudinal axis.

In air dried samples about 50 indents per collagen fibril across 2µm of each length were performed. In total, 12 and 16 collagen fibrils were tested from OIM and WT tail tendons, respectively. The mean elastic modulus per condition was determined from the mean elastic modulus per collagen fibril.

More than 20 indents in 8 and 10 PBS-hydrated collagen fibrils from OIM and WT animals, respectively (number of total fibrils tested: NOIM-♂=4, NOIM-♀=4, NWT-♂=4 and NWT-♀=6), were performed. Similarly to air-dried samples, the mean elastic modulus for each animal (per pathology per gender) was determined from the mean elastic modulus per collagen fibril.

All nanoindentation experiments were carried out under load control with maximum applied load of 150 nN for the air dried samples, 2 nN for the hydrated samples in PBS and PBS/EtOH solutions and 14 nN for the samples tested in 100% EtOH (table S1).

Table S1 Summary of the type of AFM cantilevers used, their spring constant and the applied load for each condition.

Environmental condition	AFM cantilever	Cantilever stiffness (N/m)	Applied load (nN)
Air dried	NSC15 (microMesch)	44.5 ± 5.6	150
100% PBS	PNP-TR (Nanosensors)	0.24 ± 0.02	2
PBS + EtOH	PNP-DB (Nanosensors)	0.35 ± 0.05	2
100% EtOH	AC200 (Olympus)	8.3 ± 1.8	14

PBS: phosphate buffer saline solution; EtOH: ethanol

S2 Determination of the projected area of contact

The acquisition of projected area function of contact between the indenter and the sample is required for the calculation of indentation modulus data when performing the Oliver-Pharr analysis method (Oliver and Pharr 2004).

For the NSC15 and AC200 cantilever the projected area of contact was acquired similarly to a recently proposed method (Andriotis, Manuyakorn et al. 2014). Briefly, we initially imaged a silicon spike from the calibration grating TGT1 (NT-MDT). The resulting image is an envelope image of the reference TGT1 spike and the AFM tip. Subsequently, we generate an image of the AFM tip by applying a reconstruction algorithm, programmed in Matlab 7.10.0 (R2010a), as proposed by Keller et al. (Keller and Franke 1993). We assumed the spikes to be cones with an opening angle of 50 degrees and a tip radius of 5 nm (as provided by the manufacturer). The contact area is expressed with the second order polynomial:

$$A_c(h) = ah^2 + bh \qquad \text{Eq.1}$$

where, A_c is the projected area (μm^2) at a given height h, α and b are the fitting parameters. Figure S1 shows the a three dimensional perspective of the reconstructed AFM tip and the corresponding projected area function.

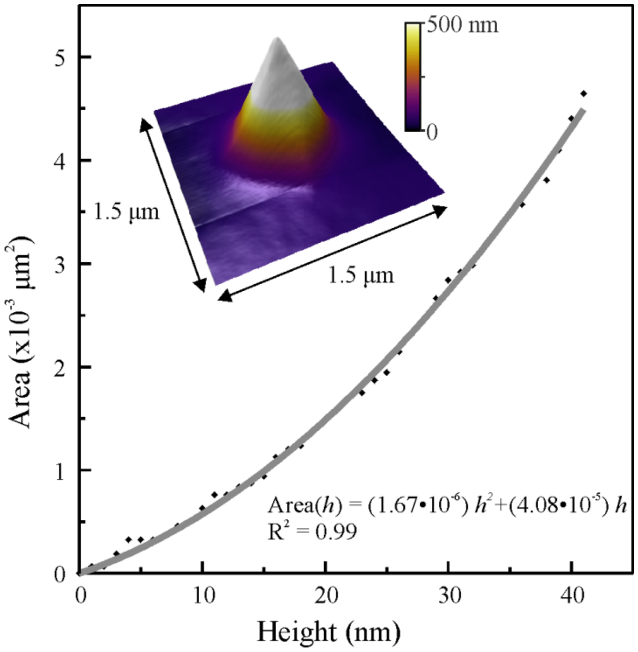

Figure S1 Typical reconstructed projected area of contact of an AFM tip. Inset shows a three dimensional perspective of the reconstructed AFM tip apex.

For the AFM cantilevers used to test the hydrated samples, i.e. the PNP-TR and PNP-DB, it was not possible to apply the aforementioned approach due to the small tip height and aspect ratio. We therefore used a geometrical approach.

The projected area of contact was estimated by assuming a pyramidal geometry (SEM images) with a round tip of radius R and half opening angle α. A graphical representation of the side view of a rounded pyramidal tip is illustrated in Figure S2. The projected area of contact, A_c, is given by:

$$A_c(h) = 4(h+\xi)^2 \tan^2 a \qquad \text{Eq.2}$$

where *h* is the indentation depth in nanometers, *ξ* is the correction distance in nanometers that accounts for the rounded tip apex and *α* is the half opening angle of the AFM tip. The correction factor *ξ* is a function of the AFM tip radius, *R,* and the half opening angle, *α*:

$$\xi(R,a) = R\left(\sin a + \cos a \cdot \tan(90 - a) - 1\right) \qquad \text{Eq.3}$$

For R=10 nm and *α*=35 degrees we have *ξ*=51.34 nm.

As illustrated in Figure S2, for indentation depths $h > R(1 - sin\,a)$ the projected area can be described by Equation 2. However, for indentation depths $h < R(1 - sin\,a)$ the projected area can be determined by assuming a spherical contact:

$$A_c(h) = \pi\left(2Rh_c - h_c^2\right) \qquad \text{Eq.4}$$

where R is the radius of the sphere as illustrated in Figure S2. Assuming a 10 nm tip radius and 35 deg half opening angle (suggested by the manufacturer), the factor *R(1-sinα)* is 4.3 nm. In this study, the minimum indentation depth during AFM cantilever-based nanoindentation with the PNP cantilevers was about 10 nm. Therefore, Equation 2 was used for the determination of the projected area of contact.

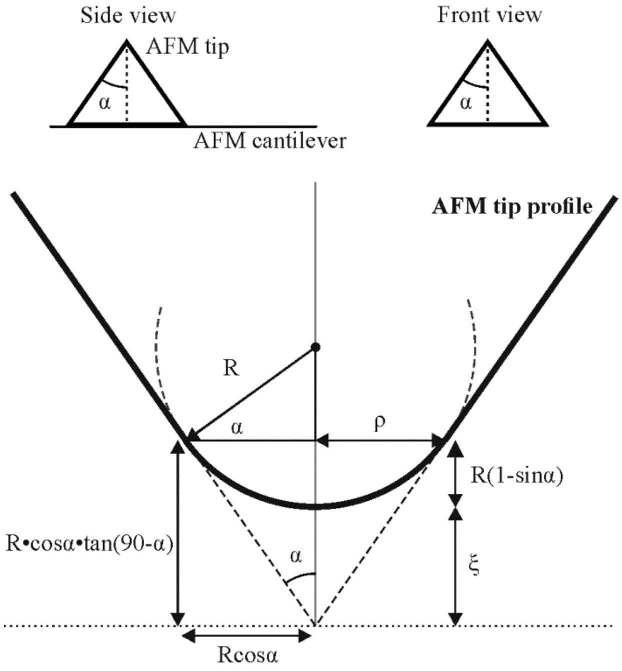

Figure S2 Determination of correction factor ξ or a 4-sided pyramidal tip with half opening angle α and tip radius R.

The contact area is finally a function of the contact depth. The contact depth was determined according to Oliver-Pharr analysis method (Oliver and Pharr 2004):

$$h_c = h_{max} - \varepsilon \frac{P_{max}}{S_c} \qquad \text{Eq.5}$$

where the ε is a constant that depends on the indenter geometry and is 0.75 for a Berkovich tip, 1 for a flat punch, and 0.73 for cone shaped indenters. The AFM tips are cone shaped indenters and thus ε was taken equal to 0.73 (Fischer-Cripps 2002, Oliver and Pharr 2004).

S3 Swelling measurements

Collagen fibrils swell, i.e. increase in fibril diameter, when hydrated with an aqueous solution, such as phosphate buffered saline solution (PBS). Swelling was measured by comparing the change in height of collagen fibrils when they were subsequently images first in air and then in PBS. For swelling measurements we assumed that only the diameter of collagen fibrils increases during hydration while their length remains unchanged. Figure S3 shows AFM images of collagen fibrils from male WT mouse dried in air (panel (a)) and hydrated in PBS (panel (b)). Panel (c) of Figure S3 compares the cross-section profiles of dry (solid line) and hydrated (dashed line) collagen fibrils from the male WT collagen fibrils while panel (d) illustrates the cross-section profiles of a male OIM collagen fibril.

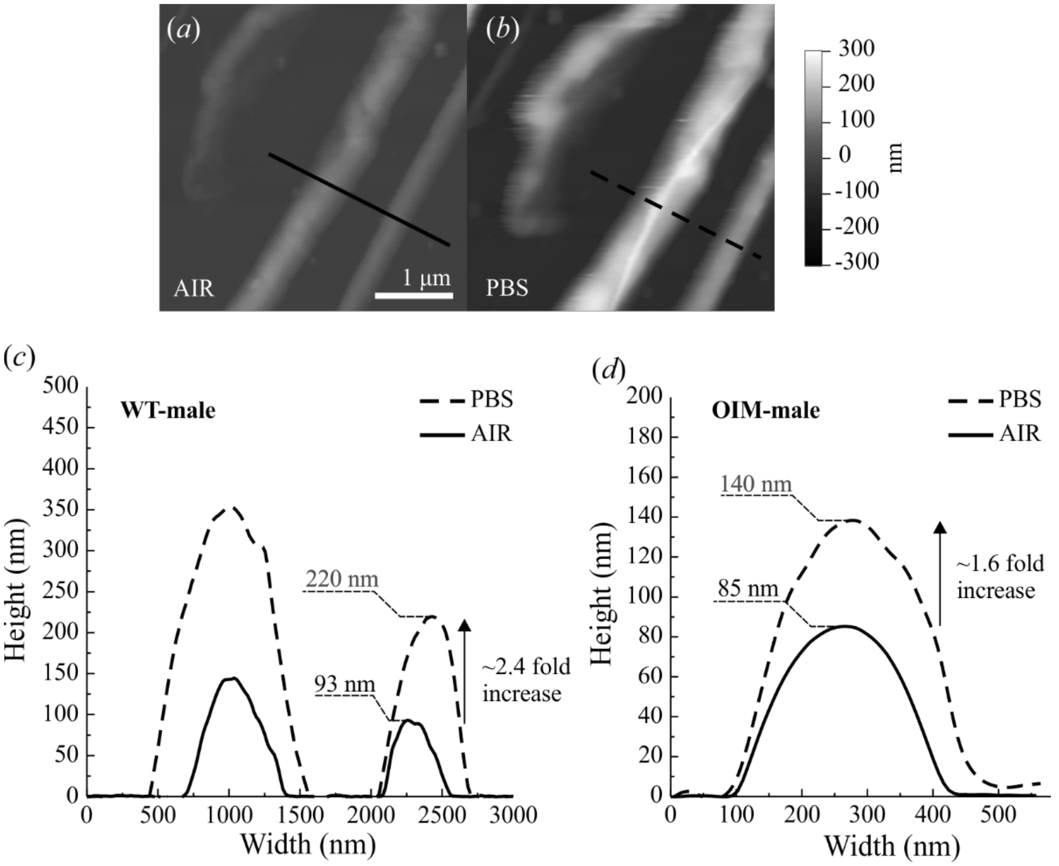

Figure S3 Swelling of collagen fibrils when hydrated with PBS. **(a)** AFM images of WT collagen fibrils imaged in air. **(b)** AFM images of WT collagen fibrils imaged in PBS. **(c)** Cross-section profiles in air (solid) and in PBS (dashed) of WT collagen fibrils. **(d)** Cross-section profiles in air (solid) and in PBS (dashed) of OIM collagen fibril

in air and in PBS. The height increases by ~2.4 times in the male WT collagen fibril and ~1.6 times in the male OIM collagen fibril.

S4 Estimation of collagen fibril density based on swelling measurements and the results gained from the *in silico* study

Assuming that the collagen fibril is geometrically characterized by a cylinder, the volume of the fibril is simply given as:

$$V = \pi \left(\frac{d}{2} \right)^2 L \qquad \text{Eq.6}$$

where *d* is the diameter and *L* is length of the fibril. Assuming the mass, m, of the fibril remains the same before and after hydration and because the swelling is described here as the fold-change in fibril diameter, we can describe the density ($\rho = m / V$) of the hydrated and the dry fibril as a function of the volume, V and therefore as a function of swelling, S:

$$\frac{\rho_{Hydrated}}{\rho_{Dry}} = \frac{V_{Dry}}{V_{Hydrated}} = \left(\frac{d_{Dry}}{d_{Wet}} \right)^2 = \left(\frac{1}{S} \right)^2 \qquad \text{Eq.7}$$

Given that the normalized volume of air-dried WT collagen fibril is $V_{Dry-WT} = 1$ and based on our *in silico* findings suggesting a 14% lower unit cell OIM volume, the air-dried OIM collagen fibril has a normalized volume $V_{Dry-OIM} = 1.14 * V_{Dry-WT}$. Assuming that the OIM and WT collagen molecules have similar weights (since they have similar number of residues; Uniprot; $M_{\alpha 1(I}$ = 138.032 kDa and $M_{\alpha 2(I)}$ = 129.557 kDa, i.e . heterotrimeric: M_{Hetero}= 405.621 kDa and M_{Homo} = 414.096 kDa yielding to 2% difference) we estimate the difference in lateral packing density between OIM and WT collagen fibrils by defining the normalized density of the dried WT collagen to be equal to 1:

$$\frac{\rho_{Dry-OIM}}{\rho_{Dry-WT}} = \frac{V_{Dry-WT}}{V_{Dry-OIM}} \qquad \text{Eq.8}$$

and therefore $\rho_{Dry-OIM} = 1.14^{-1} = 0.88$ i.e. the lateral density in the OIM collagen fibrils being 12% lower compared to the WT ones.

Then, we derive the normalized densities of the hydrated collagen fibril from Equation 7.

Therefore we write the density of the hydrated WT collagen fibrils:

$$\rho_{Hydrated-WT} = \left(\frac{1}{S_{WT}} \right)^2 \qquad \text{Eq.9}$$

and for the hydrated OIM collagen fibrils:

$$\rho_{Hydrated-OIM} = \rho_{Dry-OIM}\left(\frac{1}{S_{OIM}}\right)^2 = 0.88\left(\frac{1}{S_{OIM}}\right)^2 \qquad \text{Eq.10}$$

S5 Indentation modulus results: male vs. female animals

Figure S4 shows box plots of the indentation modulus of collagen fibrils from OIM and WT, female (♀) and male (♂) animals. The mean indentation modulus of collagen fibrils from female animals is $E_{OIM-♀-PBS}$ = (22.2 ± 3.4) MPa and $E_{WT-♀-PBS}$ = (4.0 ± 0.6) MPa and from male animals it is $E_{OIM-♂-PBS}$ = (10.3 ± 2.6) MPa and $E_{WT-♂-PBS}$ = (2.2 ± 0.6) MPa. No statistical differences were found between samples of the same health state but of different gender (P > 0.05, when using either parametric or non-parametric statistics). However, the indentation modulus of collagen fibrils from female OIM (E_{OIM}_lsmean = 21.5 MPa) was about five- fold higher compared to the one of WT (E_{WT}_lsmean = 3.7 MPa) with P < 0.001. Similarly, the indentation modulus of collagen fibrils from male OIM ($E_{OIM-♂}$_lsmean = 9.4 MPa) was about four-fold higher compared to the one of WT (E_{WT}_lsmean = 1.9 MPa) with P < 0.001.

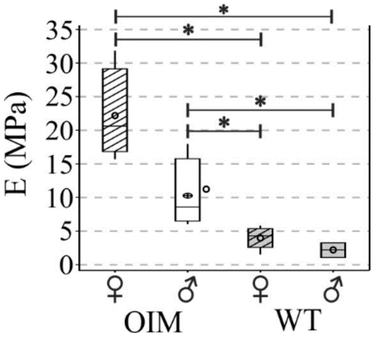

Figure S4 Box plots of indentation modulus results comparing male vs. female animals.

References

Andriotis, O. G., W. Manuyakorn, J. Zekonyte, O. L. Katsamenis, S. Fabri, P. H. Howarth, D. E. Davies and P. J. Thurner (2014). "Nanomechanical assessment of human and murine collagen fibrils via atomic force microscopy cantilever-based nanoindentation." Journal of the mechanical behavior of biomedical materials **39**: 9-26.
Fischer-Cripps, A. (2002). Nanoindentation, Springer.
Keller, D. J. and F. S. Franke (1993). "Envelope reconstruction of probe microscope images." Surface Science **294**(3): 409-419.
Oliver, W. and G. Pharr (2004). "Measurement of hardness and elastic modulus by instrumented indentation: Advances in understanding and refinements to methodology." J. Mater. Res **19**(1): 3-20.
