# Supplementary material for: Structure–mechanics relationships of collagen fibrils in the osteogenesis imperfecta mouse model
Source: J R Soc Interface. 2015 Oct 6;12(111):20150701. doi: 10.1098/rsif.2015.0701 (PMC4614505; doi:10.1098/rsif.2015.0701)
Supplement: Table S2-ESM [file rsif20150701supp2.pdf]

Table S2 Unit cell dimensions of WT and OIM collagen microfibril model at 1atm and 75 MPa applied stress.

		Unit cell dimensions (A)	
Stress		*a*	*b*
1 atm	WT	33.41	25.08
	OIM	36.16	25.54
	Ratio (WT/OIM)	0.924	0.982
75 MPa	WT	29	25.44
	OIM	31.03	25.87
	Ratio (WT/OIM)	0.935	0.983
