# Supplementary material for: Structure–mechanics relationships of collagen fibrils in the osteogenesis imperfecta mouse model
Source: J R Soc Interface. 2015 Oct 6;12(111):20150701. doi: 10.1098/rsif.2015.0701 (PMC4614505; doi:10.1098/rsif.2015.0701)
Supplement: Figure S5-chemical dehydration [file rsif20150701supp3.pdf]

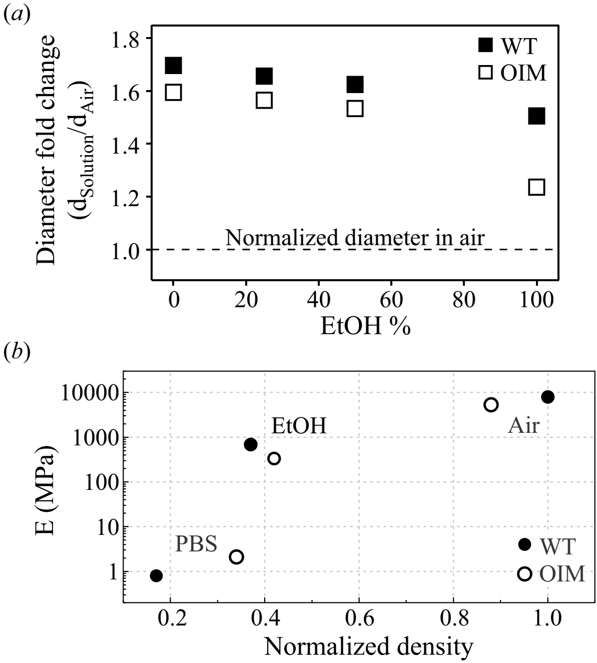

Figure S5 (a) Fold change in the diameter of WT and OIM collagen fibrils during chemical dehydration. The fold change is presented in respect to the fibril diameter in air. **(b)** Indentation modulus versus the normalized density of PBS-hydrated, EtOH-dehydrated and air-dried WT and OIM collagen fibrils.
